# Metabolic and hormonal responses to acute high-load resistance exercise in normobaric hypoxia using a saturation clamp

**DOI:** 10.3389/fphys.2024.1445229

**Published:** 2024-09-02

**Authors:** Guole Jiang, Shuang Qin, Bing Yan, Olivier Girard

**Affiliations:** ^1^ Sports Coaching College, Beijing Sport University, Beijing, China; ^2^ College of Basic Military and Political Education, National University of Defense Technology, Changsha, China; ^3^ School of Sport Medicine and Rehabilitation, Beijing Sport University, Beijing, China; ^4^ College of Acupuncture and Tuina and Rehabilitation, Hunan University of Chinese Medicine, Changsha, China; ^5^ China Institute of Sport and Health Science, Beijing Sport University, Beijing, China; ^6^ School of Human Sciences (Exercise and Sport Science), University of Western Australia, Perth, WA, Australia

**Keywords:** systemic hypoxia, simulated altitude, strength, endocrine responses, anabolic hormones

## Abstract

**Introduction:**

We assessed metabolic and hormonal responses to high-load resistance exercise under varying normobaric hypoxia conditions with a saturation clamp.

**Methods:**

Employing a counterbalanced, crossover test design, ten well-trained men participated in three exercise trials with normoxic or hypoxic gas mixtures to maintain arterial oxygen saturation at −90% and 80% [moderate (MH) and severe (SH) hypoxia, respectively]. The resistance exercise regimen comprised five sets of 10 repetitions of barbell back squats at 70% of one repetition maximum, with 1-min rest between sets. Metabolic and hormonal responses were measured before normoxia or hypoxia exposures (Pre 1), 15 min after the exposures (Pre 2), and at 0-, 15-, and 30-min post-exercises (T0, T15, and T30, respectively).

**Results:**

Compared to Pre 2, blood lactate concentrations and growth hormone values were elevated at T0, T15, and T30 (*p* ≤ 0.001), while testosterone values increased at T0 in all conditions (*p* ≤ 0.009). Epinephrine values increased significantly from Pre 2 to T0 in SH only (*p* < 0.001). SH had significantly higher blood lactate concentrations (*p* = 0.023), growth hormone (*p* = 0.050), and epinephrine (*p* = 0.020) values at T30 compared to NM. Cortisol values were elevated above Pre 2 at T15 in MH and SH, while lower testosterone values were noted at T0 and T15 for SH compared to NM and MH (all *p* ≤ 0.05).

**Discussion:**

Severe simulated hypoxia, achieved through a saturation clamp during barbell back squats, may enhance metabolic and hormonal responses, particularly 30 min post-session. Nevertheless, the acute effects of hypoxia exposure seem to be overridden by the impact of high-load resistance exercise.

## Introduction

Engaging in resistance exercise under systemic hypoxia (RTH), with a reduced inspired oxygen fraction (FiO_2_ 0.120–0.160), is a popular intervention for improving muscular strength, hypertrophy, and overall health (Ramos-Campo et al., 2018). Pioneers like Nishimura et al. (2020) demonstrated greater improvements in muscle cross-sectional area and faster strength gains with 6-week moderate-intensity resistance training (70% of one repetition maximum) in hypoxia (FiO_2_ 0.160) compared to normoxia. Subsequent studies (Manimmanakorn et al., 2013; Innes et al., 2016; Yan et al., 2016) support these benefits, although not all research consistently reports such advantages (Ho et al., 2014; Törpel et al., 2020). Limited oxygen availability, leading to metabolic accumulation and cell swelling, is believed to preferentially recruit type II muscle fibers, potentially influencing training adaptations (Scott et al., 2016). Moreover, acute hypoxia, as a stressor, can elevate the production of hormones crucial for regulating metabolic and anabolic processes (Athanasiou et al., 2023).

Research on endocrine responses to acute resistance exercise in hypoxia has produced mixed findings. Some studies report higher levels of lactate, growth hormone and/or cortisol during the 0–60 min post-exercise period in hypoxia compared to normoxia (Kon et al., 2010; Kon et al., 2012; Filopoulos et al., 2017), while others show no significant differences (Ho et al., 2014). These inconsistencies may stem from differences in resistance training protocols such as the load lifted and exercise structure, as well as the range of FiO_2_ levels applied (Timon et al., 2022). Exposure to systemic hypoxia introduces notable inter-individual variability (Chapman et al., 1998). For example, exposure to extreme hypoxia (FiO_2_ 0.110) for 4 h led to a significant decrease in arterial oxygen saturation (SpO_2_) in all participants, with individual SpO_2_ drops ranging from 12% to 36% (D’Hulst et al., 2013). Consequently, relying on a fixed FiO_2_ as a marker of hypoxic dose may result in considerable inter-individual variability in metabolic and hormonal hypoxia responses (Soo et al., 2020).

A solution to this problem is the saturation clamp approach (Soo et al., 2020), which requires adjusting the FiO_2_ for each individual to maintain SpO_2_ at a target level. This method has been successfully employed in RTH literature. For instance, Manimmanakorn et al. (2013) implemented a 5-week training RTH program for netball athletes, employing low-loads (thrice weekly, three sets of knee extension and flexion to failure at 20% 1RM, FiO_2_ adjusted to maintain SpO_2_ at −80%). The RTH group exhibited a higher increase in repetitions at 20% 1RM compared to the normoxic training control. While most studies using a saturation clamp have focused on comparing physical performance, erythropoietin expression, and body composition between young and older adults using a single hypoxic dose of ∼80–85% (Törpel et al., 2019; Törpel et al., 2020), this approach has not been widely applied to investigate acute metabolic and hormonal responses to resistance exercise in varying levels of hypoxia.

The aim of this study was to evaluate the acute metabolic and hormonal responses to high-load resistance exercise under varying levels of normobaric hypoxia, employing a SpO_2_ clamp approach. We hypothesized that the most severe hypoxic condition would lead to increased blood lactate accumulation and anabolic hormone responses.

## Methods

### Participants

Ten well-trained men (mean age 21.2 ± 2.3 years; height 178.4 ± 2.7 cm; body weight 69.8 ± 6.3 kg), all experienced in resistance exercise, participated in the study. Participants were recruited from local sporting clubs and had a minimum of 2 years of resistance training experience. They were classified as “Trained/Development” (Tier 2) using established criteria (McKay et al., 2022). Throughout the testing phase, none of the participants were using substances (e.g., anabolic steroids, creatine, sympathoadrenal drugs) that could potentially impact the study results. During the study, participants resided at sea level in Beijing (China), and reported no exposure to altitudes above 1,500 m within 6 months before the experimental trials. The study was approved by Beijing Sport University Review Board for Human Participants (no. 2017004A), with written informed consent obtained from participants.

### Study design

The study employed a single-blind, counterbalanced, crossover test design. Initially, participants underwent a preliminary session in normoxia, where they familiarized themselves with the correct technique for performing a barbell back squat and determined their one-repetition maximum mean (139.8 ± 16.3 kg) (Yan et al., 2016). Subsequently, participants completed three separate experimental sessions, during which they performed barbell back squats while inhaling either a normoxic (normoxia) or hypoxic gas mixture. In the hypoxic conditions, SpO_2_ was controlled at −90% and 80% for moderate (MH) and severe hypoxia (SH), respectively. Each session was separated by at least 2 weeks and conducted at the same time of day (8.30–11.30 a.m.) for each participant.

### Experimental sessions

After an overnight fast, participants arrived at the laboratory and rested in a seated position for 30 min before the first blood collection (Pre 1) outside the environmental chamber while breathing room air (i.e., before exposure to normoxic or hypoxic gas mixtures). They then entered the environmental chamber, rested (i.e., sitting position) for an additional 15 min with exposure to either normoxic or hypoxic gas, and had the second capillary blood sample taken (Pre 2). Afterwards, participants commenced with a warm-up set of 10 repetitions of barbell back squats at 40% of 1RM, followed by the main exercise consisting of five sets of 10 repetitions of barbell back squats at 70% of 1RM (97.8 ± 11.4 kg), with a 1-min rest period between sets. During the squats, they were instructed to achieve a knee angle of 90° and were assisted if they experienced fatigue during the final repetitions. Three additional blood samples were collected immediately (T0), 15 (T15), and 30 min (T30) after completing the resistance exercise. After the initial blood sample (Pre 1), participants remained in the testing environment until the final blood sample was collected. The exposures continued until the experimental trial ended, at which points participants left the environmental chamber. Participants could consume water *ad libitum* throughout the session.

### Saturation clamping

Participants were exposed to either normoxic or normobaric hypoxic air in an environmental chamber (L.O.S. LOWOXYGEN SYSTEMS GmbH, Germany). The “background” FiO_2_ inside the chamber was set at 0.209 for normoxic air (NM) and 0.166 for both MH and SH conditions. Participants also inhaled a gas mixture delivered by a hypoxic generator (Hypoxico, Inc. New York, United States), as adjusting FiO_2_ from this device has a faster response rate on physiological markers including SpO_2_ than an environmental chamber. Instead of fixing a specific simulated altitude or FiO_2_ for the hypoxic conditions, SpO_2_ was clamped at −90% and −80% in MH and SH, respectively. This was achieved by continuously adjusting the FiO_2_ individually during each trial, following a manual procedure that was piloted in advance. Continuous monitoring of SpO_2_ was conducted using a pulse oximeter (WristOx23150; Nonin Medical, Inc., United States) placed on the forefinger of each participant. For blinding purposes, participants consistently breathed through the same set-up, including during normoxia.

### Blood sampling and analysis

Blood samples were drawn from an antecubital vein through an intravenous catheter at several time points: in normoxia (Pre 1), 15 min after exposure to either normoxia or hypoxia at rest (Pre 2), as well as immediately after, 15 and 30 min after exercise (T0, T15 and T30, respectively). Participants remained in the testing environment until the final blood sample was collected. After collection, the serum was separated by centrifugation at 5,000 rpm for 5 min and stored at −80°C until analysis. The storage time from collection to analysis was within 72 h.

Commercial test kits were used to measure the concentrations of growth hormone (Access Ultrasensitive hGH assay; Catalog No. 33580; Beckman Coulter, Inc., Brea, CA, United States), testosterone (Access Testosterone; Catalog No. 33560; Beckman Coulter, Inc., Brea, CA, United States) and cortisol (Access Cortisol; Catalog No. 33600; Beckman Coulter, Inc., Brea, CA, United States) using an automatic immunoassay system (Unicel DXI 800; Beckman Coulter, Inc., United States). The epinephrine concentration was determined using an enzyme immunoassay kit (CatCombi ELISA; Catalog No. RE59242; IBL International GmbH, Germany), while blood lactate concentration was measured using an automatic lactate analyzer (Biosen C-line; EKF diagnostic GmbH, Germany).

### Statistical analysis

All statistical analyses were conducted using IBM SPSS Statistics for Windows, version 25.0 (IBM Corp., Armonk, NY, United States). Descriptive statistics are presented as mean ± SD. To evaluate the effects of high-load resistance exercise under varying normobaric hypoxia conditions on metabolic and hormonal responses, we employed generalized estimating equations (GEE) for each outcome variable. GEE was chosen for its robustness in handling repeated measures and correlated data inherent in our crossover study design. Each GEE model included a single metabolic or hormonal response variable as the dependent variable, with oxygen condition (NM, MH, and SH), time points (Pre 1, Pre 2, T0, T15, and T30), and their interaction as the main independent variables. An exchangeable correlation structure was used to account for repeated measures within participants, assuming equal correlation between all time points. The Pre 2 value was included as a covariate to control for its influence on the outcome variables. To assess the effectiveness of the SpO_2_ clamping procedure, we analyzed SpO_2_ data using a GEE model with SpO_2_ as the dependent variable, and the same independent variables (oxygen condition and time points) and their interaction. The Pre 1 value was included as a covariate. An exchangeable correlation structure was also applied. Post-hoc analyses were performed using the least significant difference (LSD) method to adjust for multiple comparisons following the GEE analysis. Statistical significance was set at *p* ≤ 0.05.

## Results

**FIGURE 1:**
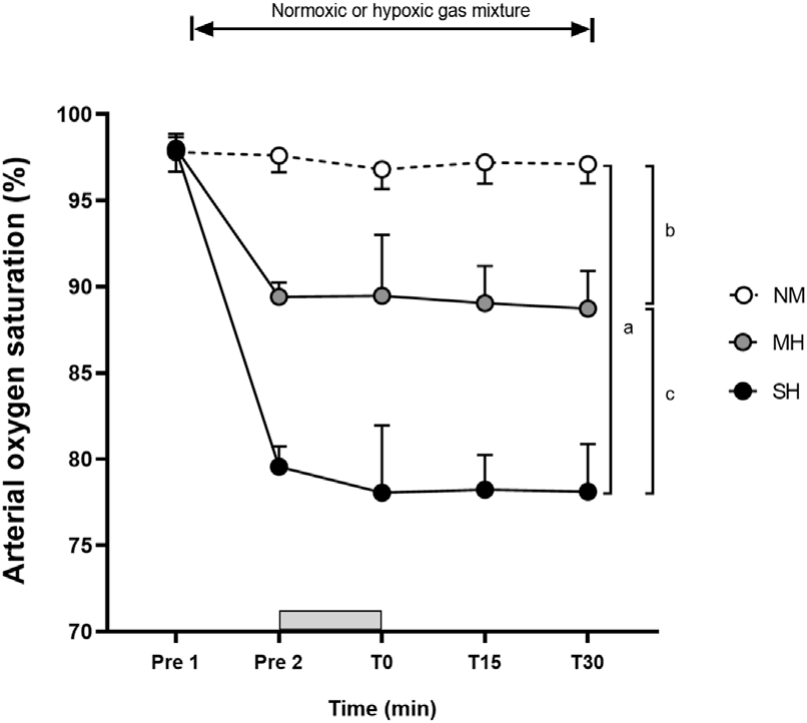
Arterial oxygen saturation levels before and following resistance exercise while inhaling either normoxic (NM) or hypoxic gas mixtures with controlled arterial oxygen saturation of −90% and 80% for moderate (MH) and severe hypoxia (SH), respectively. Mean ± SD (n = 10). Arterial oxygen saturation was obtained before exposure to normoxia or hypoxia (Pre 1), 15 min after exposure (Pre 2), as well as immediately after, 15 and 30 min after exercise (T0, T15, and T30, respectively), while participants remained in the testing environment. The Gy bar indicates the resistance exercise period. ^a^, ^b^, and ^c^ significant difference NM vs. SH, NM vs. MH, and MH vs. SH, respectively (*p* < 0.05).

**FIGURE 2:**
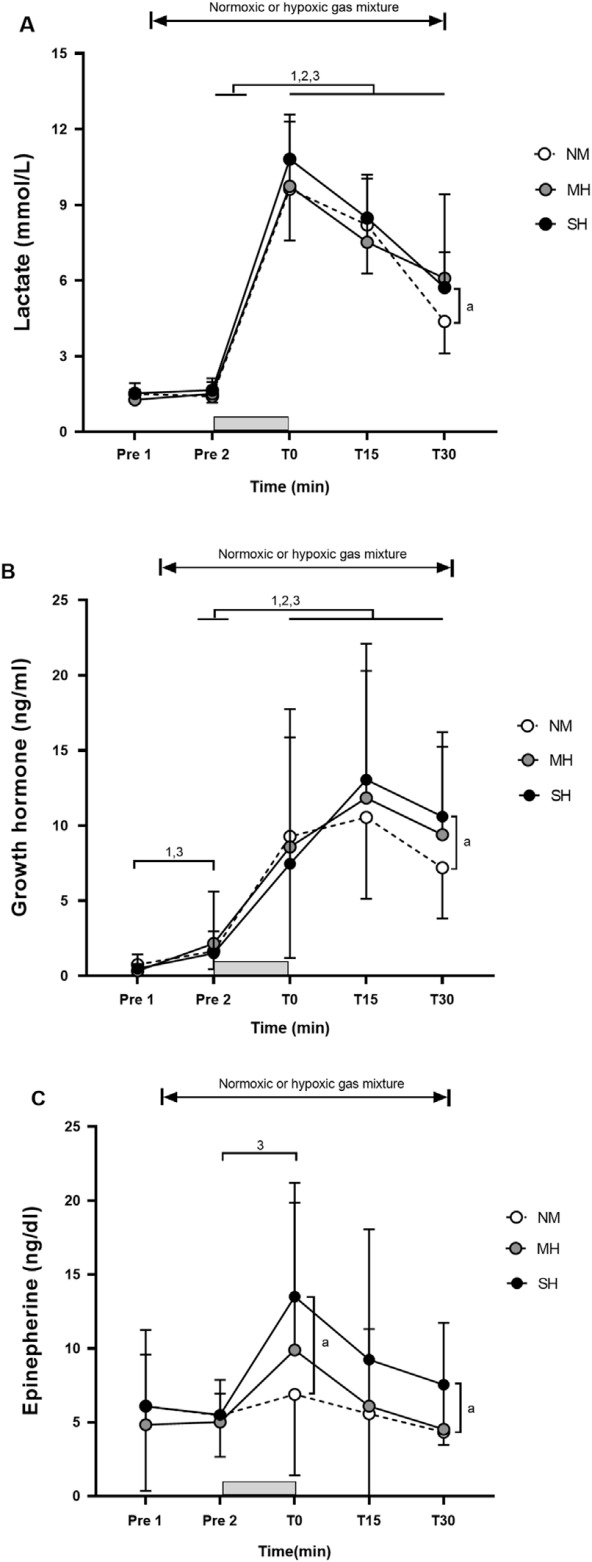
Blood lactate concentration **(A)**, growth hormone **(B)**, and epinephrine **(C)** before and following resistance exercise while inhaling either normoxic (NM) or hypoxic gas mixtures with controlled arterial oxygen saturation of −90% and 80% for moderate (MH) and severe hypoxia (SH), respectively. Mean±SD (n = 10). Arterial oxygen saturation was obtained before exposure to normoxia or hypoxia (Pre 1), 15 min after exposure (Pre 2), as well as immediately after, 15 and 30 min after exercise (T0, T15, and T30, respectively), while participants remained in the testing environment. The gray bar indicates the resistance exercise period. ^1^, ^2^, and ^3^ significantly different between time points for NM, MH and SH, respectively (*p* < .05). ^a^ significant difference NM vs. SH (*p* < 0.05).

**FIGURE 3:**
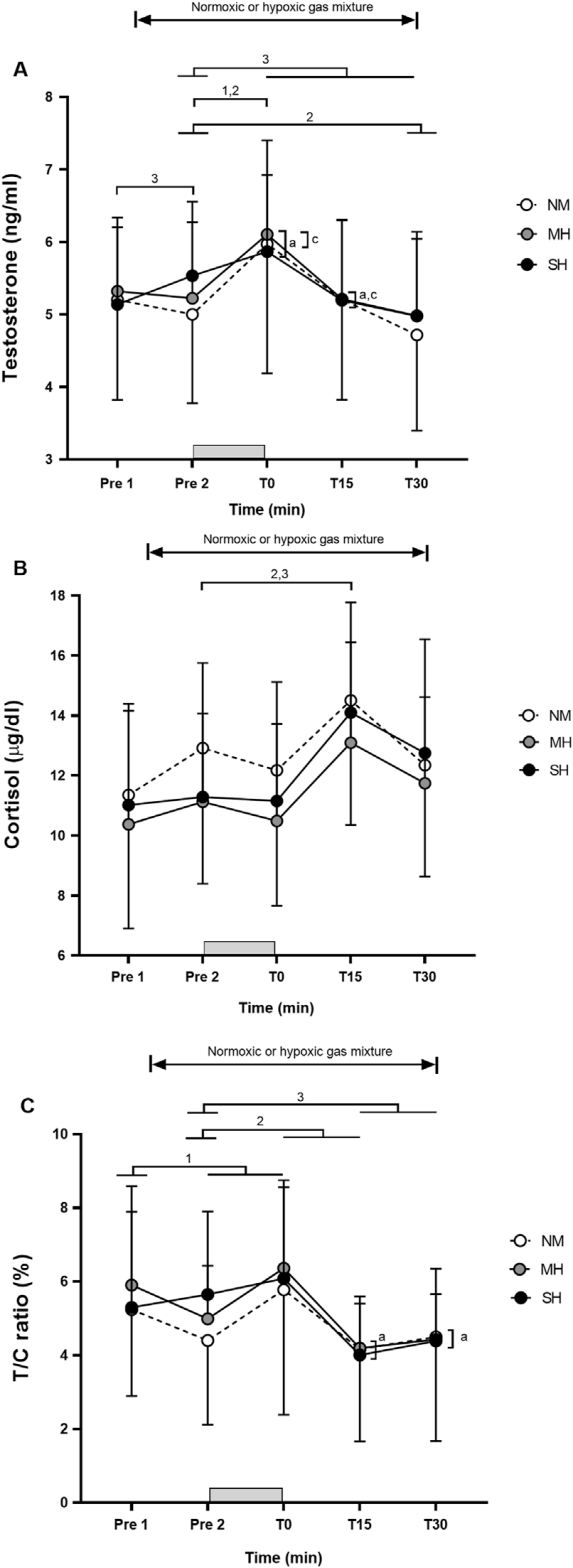
Testosterone **(A)**, cortisol **(B)**, and testosterone/cortisol ratio **(C)** before and following resistance exercise while inhaling either normoxic (NM) or hypoxic gas mixtures with controlled arterial oxygen saturation of −90% and 80% for moderate (MH) and severe hypoxia (SH), respectively. Mean ± SD (n = 10). Arterial oxygen saturation was obtained before exposure to normoxia or hypoxia (Pre 1), 15 min after exposure (Pre 2), as well as immediately after, 15 and 30 min after exercise (T0, T15, and T30, respectively), while participants remained in the testing environment. The gray bar indicates the resistance exercise period. ^1^, ^2^, and ^3^ significantly different between time points for NM, MH and SH, respectively (*p* < 0.05). ^a^ and ^c^ significant difference NM vs. SH and MH vs. SH, respectively (*p* < 0.05).

Figure 1 demonstrates the effectiveness of the SpO_2_ clamping procedure. Compared to Pre 2, blood lactate concentrations and growth hormone values were elevated at T0, T15, and T30 (*p* ≤ 0.001; Figure 2), while testosterone values increased at T0 in all conditions (*p* ≤ 0.009; Figure 3). Epinephrine values increased significantly from Pre 2 to T0 in SH only (*p* < 0.001). Additionally, SH had significantly higher blood lactate concentrations (*p* = 0.023), growth hormone (*p* = 0.050), and epinephrine (*p* = 0.020) values at T30 compared to NM. Cortisol values were elevated above Pre 2 at T15 in MH (*p* = 0.007) and SH (*p* = 0.034). Lower testosterone values were noted at T0 and T15 for SH compared to NM and MH (*p* ≤ 0.023).

## Discussion

### High-load resistance exercise supersedes the acute effects of hypoxia exposure

In partial agreement with our hypothesis, it was observed that SH, but not MH, during high-load resistance exercise using a saturation clamp may slightly enhance metabolic and hormonal responses, particularly 30 min post-session, compared to normoxia. This finding occurred despite employing brief inter-set rest periods (1 min), high-load resistance exercise (70% of one repetition maximum) that recruited large muscle mass (barbell back squats), substantial exercise volume (five sets of 10 repetitions), and providing a significant hypoxic stimulus for all participants (Ramos-Campo et al., 2018). Notably, resting plasma growth hormone levels were previously found to increase only following hypoxic resistance training in severe hypoxia (FiO_2_ 0.136), with no such increase noted in moderate hypoxia (FiO_2_ 0.158) or normoxia (Namboonlue et al., 2020). Our findings align with the study by Ho et al. (2014), who reported that low-intensity squat exercise (30% of 1RM) performed under mild simulated hypoxia (FiO_2_ 0.150; SpO_2_ −92%) did not induce significantly greater hormonal responses in growth hormone, total testosterone, and cortisol than normoxia. However, other studies have shown that performing bench press and leg press at 50%–85% of 1RM in severe normobaric hypoxic conditions (FiO_2_ 0.130; SpO_2_ ∼82–85%) induced larger blood lactate accumulation as well as greater growth hormone and epinephrine responses than in the normoxic condition. Interestingly, other anabolic hormones such as testosterone or cortisol remained unchanged (Kon et al., 2010; Kon et al., 2012). Despite achieving consistent SpO_2_ values between conditions through our saturation clamp approach, high-load resistance exercise likely supersedes the acute effects of hypoxia exposure in well-trained men.

### Concomitance between metabolic and hormonal responses

Overall, the magnitude and time course of changes in metabolic and hormonal responses during the 30-min post-exercise period were consistent with prior studies using fixed FiO_2_ levels ranging from 0.126 to 0.160 (Kon et al., 2010; Kon et al., 2012; Yan et al., 2016). The stimulation of growth hormone secretion during exercise is believed to be influenced by the accumulation of metabolic by-products, such as lactate or H^+^ (Gordon et al., 1994). The acidic muscle environment may stimulate sympathetic nerve activity through chemoreceptive reflexes (Victor and Seals, 1989), leading to increases in growth hormone, norepinephrine, and lactate levels (Buresh et al., 2009). However, in several studies (Kurobe et al., 2015; Yan et al., 2016; Namboonlue et al., 2020), changes in blood lactate concentrations did not fully explain the observed changes in growth hormone. Resting plasma growth hormone levels, for instance, were elevated only following hypoxic resistance training in severe hypoxia (FiO_2_ 0.136), with no increase observed in moderate hypoxia (FiO_2_ 0.158) or in normoxia (Namboonlue et al., 2020). In our study, blood lactate concentrations peaked at T0, while growth hormone values were highest at T15 in all conditions. Resistance training predominantly targets type II muscle fibers, associated with a higher release of lactic acid and growth hormone (Schoenfeld, 2010). However, the mechanisms underlying growth hormone production and release during and after hypoxic resistance training remain uncertain, and the precise role of growth hormone in this context is not fully understood. The significantly higher blood lactate concentrations, growth hormone, and epinephrine values at T30 in SH compared to NM in our study may suggest an alteration in muscle fiber recruitment (shifting from predominantly Type I to Type II) in the more severe hypoxic condition. This indirectly supports the notion of a slightly heightened anabolic response associated with RTH compared to equivalent training in normoxia due to a greater accumulation of metabolic by-products (Scott et al., 2015a).

### Variability in metabolic and hormonal responses

Despite controlling SpO_2_ experimentally, participants showed significant variability in hormonal responses to acute resistance exercise, with or without hypoxia. This variability in post-exercise blood lactate concentration, growth hormone, and other hormonal responses across all time points and conditions is likely influenced by factors beyond hypoxia exposure. This is reinforced by the observation that growth hormone increased significantly from Pre 1 to Pre 2 in both SH and NM conditions, but not in MH; however, the changes with 15 min of passive hypoxic exposure are likely not clinically relevant. Testosterone increased only in SH. Higher, though not statistically significant, testosterone values were previously observed 15 min after exposure to both normoxic and hypoxic (FiO_2_ 0.130) mixtures (Kon et al., 2012). Individual differences in hypoxic tolerance, genetics, and baseline fitness levels are probably contributors (Deldicque, 2022). Practitioners should recognize substantial between-individual differences in both the magnitude of adjustments to a resistance exercise protocol and responses to a given hypoxic stimulus. While the exact cause of this variability is beyond the scope of this study, our results underscore that the potential for hypoxia to enhance hormonal responses to resistance exercise is highly individualized, with some participants exhibiting stronger responses than others, even with a saturation clamp approach.

### Arterial oxygen saturation

In our study, employing a saturation clamp, hypoxic exposure increased SpO_2_ response variability between participants compared to NM, particularly at T0, and to a lesser extent at both T15 and T30. Visual inspection of Figure 1 also suggests a comparable magnitude between MH and SH. Taken together, this suggests that exercise, in addition to hypoxia exposure, might have influenced SpO_2_ readings, especially when monitored from the finger where blood flow may have been limited during squatting. During back squat and deadlift exercises, prior observations noted SpO_2_ dropping from 95% in normoxia to −90% in moderate hypoxia (FiO_2_ 0.160) and −75% in severe hypoxia (FiO_2_ 0.130), with greater variability observed in the latter (Scott et al., 2015b). Similar to this study, the prevailing method for controlling SpO_2_ during hypoxic exposure involves manual adjustments to the individual’s FiO_2_ (Mira et al., 2021). One limitation is that the frequency and magnitude of manual adjustments in FiO_2_ depend on the discretion of investigators, introducing potential inter-rater reliability issues. To eliminate potential inter-rater differences from the manual approach, FiO_2_ adjustments were conducted by a single experimenter familiar with the equipment to minimize bias. With technological advancements, automatic FiO_2_ adjustments for SpO_2_ clamping are likely to be more reliable and accurate. Supporting this, automatic methods for heart rate clamping are more accurate than manual approaches (e.g., power output adjustments every 30 s by 0.5 or 10 W) during submaximal intensity continuous cycling (Li et al., 2024). Others have employed automatic saturation clamps by the hypoxicator using a biofeedback control system (Manimmanakorn et al., 2013; Törpel et al., 2019; Törpel et al., 2020), but data about the accuracy and reliability of this practice are lacking. Therefore, a more robust approach involving real-time and continuous adjustments to SpO_2_ is needed for achieving precise saturation clamps that can be practically applied.

### Limitations and additional considerations

Several methodological considerations should be taken into account when interpreting our findings. Firstly, with the exception of one study in professional rugby players (Mayo et al., 2018) and one involving female netballers (Manimmanakorn et al., 2013), all RTH studies conducted so far focused on untrained participants or recreationally trained athletes (Deldicque, 2022). This limits the generalizability of our findings, particularly to individuals with different demographics, where gender differences in the endocrine and metabolic responses to hypoxic exercise have been noted (Raberin et al., 2023).

Secondly, substantial heterogeneity exists in the applied exercise stimulus across the RTH literature, encompassing differences in lifting velocity, load, and rest periods. For instance, benefits have been observed using loads ranging from 20% to 90% of 1RM (Deldicque, 2022; Benavente et al., 2023). Our study, like some others, utilized a moderate-load hypertrophy-based training prescription (e.g., 4 sets of 10 repetitions with 70% 1RM and 60 s rest; Nishimura et al., 2020). On the other hand, other studies incorporated heavy strength training sessions focusing on maximal strength (e.g., 2–4 sets of 3–6 repetitions with >75% 1RM and 180 s inter-set rest; Innes et al., 2016). There are also instances of light-load exercises seemingly targetting muscular endurance development (e.g., 3 sets to failure with 20% 1RM and 30 s rest; Manimmanakorn et al., 2013). Future studies employing different exercise structures should investigate whether more severe hypoxia could acutely enhance metabolic and hormonal responses compared to lower severities or normoxia. Another key methodological consideration is that our design, based on previous studies (Kon et al., 2012; Kon et al., 2021), required participants to remain in the chamber post-exercise, facilitating comparison with existing literature. Different hormonal responses might have occurred if participants had not stayed in the environment for 30 min after exercise until the final blood sample was collected. Caution is needed when generalizing our findings to conditions where the post-RTH recovery period is consistently performed in normoxia.

Thirdly, a crucial aspect in designing RTH programs is the metabolic stress created (Deldicque, 2022). Hypoxia fundamentally reduces oxygen availability, acutely altering metabolic processes essential for energy production. Due to the absence of near-infrared spectroscopy measurements in our study, it is challenging to ascertain whether severer hypoxia severities are more effective in causing a greater decline in muscle oxygenation during resistance exercise (Girard et al., 2022). Importantly, Benavente et al. (2023) found no significant effects of moderate (FiO_2_ 0.143–0.160) *versus* severe (FiO_2_ = <0.142–0.110) hypoxia on RTH efficacy. Therefore, exercise programs should be tailored to leverage the metabolic challenge posed by hypoxia. While our study only modified SpO_2_ levels, practitioners should always consider the hypoxic dose along with the exercise stimulus demands when planning RTH programs. While beneficial effects of hypoxia over normoxia have been found with intensities ranging from 20% to 90% of 1-RM, moderate-load exercise (70% of 1-RM) with inter-set rest periods of 1 min and FiO_2_ inducing SpO_2_ levels ranging from 80% to 90% follows current recommendations for structuring RTH workouts (Deldicque, 2022). Considering exercise to failure as an additional variable, distinct from other exercise structure elements (e.g., inter-set recovery period, load lifted) or the severity of hypoxia, may amplify metabolic stress. It can not be ruled out that performing the final set or sets of the back squat exercise to failure could have generated additional metabolic stress (Deldicque, 2022), potentially resulting in more pronounced differences between conditions.

Finally, the implementation of high-load circuit-style resistance training in hypoxia emerges a promising RTH approach (Martinez-Guardado et al., 2019; Ramos-Campo et al., 2018). However, this intervention encounters challenges, especially when training large groups simultaneously in a climatic chamber or hypoxic tent. To date, the practice of surreptitiously adjusting FiO_2_ levels for a consistent reduction in SpO_2_ requires participants to wear a facemask connected to a hypoxicator. Therefore, the application of RTH using a saturation clamp is currently confined to individuals executing isolated localized or whole-body movements. Analogous to the automatic heart rate clamp approach during aerobic exercise (Li et al., 2023), technological development is essential to automatically control the internal load of resistance exercise (i.e., SpO_2_ levels or muscle oxygenation status using near-infrared spectroscopy) across various hypoxic conditions.

## Conclusion

Achieving sever hypoxia through a saturation clamp during barbell back squats may enhance metabolic and hormonal responses, especially 30 min after the session. Nevertheless, the impact of high-load resistance exercise appears to surpass the acute effects of hypoxia exposure in well-trained men.

## Data Availability

The raw data supporting the conclusions of this article will be made available by the authors, without undue reservation.
